# Necroptosis inhibition counteracts neurodegeneration, memory decline, and key hallmarks of aging, promoting brain rejuvenation

**DOI:** 10.1111/acel.13814

**Published:** 2023-03-27

**Authors:** Macarena S. Arrázola, Matías Lira, Felipe Véliz‐Valverde, Gabriel Quiroz, Somya Iqbal, Samantha L. Eaton, Douglas J. Lamont, Hernán Huerta, Gonzalo Ureta, Sebastián Bernales, J. César Cárdenas, Waldo Cerpa, Thomas M. Wishart, Felipe A. Court

**Affiliations:** ^1^ Center for Integrative Biology, Faculty of Sciences Universidad Mayor Santiago Chile; ^2^ Geroscience Center for Brain Health and Metabolism (GERO) Santiago Chile; ^3^ Departamento de Biología Celular y Molecular, Facultad de Ciencias Biológicas Pontificia Universidad Católica de Chile Santiago Chile; ^4^ The Roslin Institute University of Edinburgh Edinburgh UK; ^5^ Massive Analytic Ltd. London UK; ^6^ FingerPrints Proteomics Facility School of Life Sciences, University of Dundee Dundee UK; ^7^ Fundación Ciencia & Vida Santiago Chile; ^8^ Buck Institute for Research on Aging Novato California USA; ^9^ Department of Chemistry and Biochemistry University of California Santa Barbara California USA

**Keywords:** aging, axon pathology, cognition, hippocampus, memory, necroptosis, rejuvenation, synaptic transmission

## Abstract

Age is the main risk factor for the development of neurodegenerative diseases. In the aged brain, axonal degeneration is an early pathological event, preceding neuronal dysfunction, and cognitive disabilities in humans, primates, rodents, and invertebrates. Necroptosis mediates degeneration of injured axons, but whether necroptosis triggers neurodegeneration and cognitive impairment along aging is unknown. Here, we show that the loss of the necroptotic effector *Mlkl* was sufficient to delay age‐associated axonal degeneration and neuroinflammation, protecting against decreased synaptic transmission and memory decline in aged mice. Moreover, short‐term pharmacologic inhibition of necroptosis targeting RIPK3 in aged mice, reverted structural and functional hippocampal impairment, both at the electrophysiological and behavioral level. Finally, a quantitative proteomic analysis revealed that necroptosis inhibition leads to an overall improvement of the aged hippocampal proteome, including a subclass of molecular biofunctions associated with brain rejuvenation, such as long‐term potentiation and synaptic plasticity. Our results demonstrate that necroptosis contributes to age‐dependent brain degeneration, disturbing hippocampal neuronal connectivity, and cognitive function. Therefore, necroptosis inhibition constitutes a potential geroprotective strategy to treat age‐related disabilities associated with memory impairment and cognitive decline.

## Graphical abstract

Necroptosis is activated along brain aging, and is associated with the deterioration of axons and synaptic function, increasing neuroinflammation and affecting cognitive processes, such as learning and memory. Remarkably, the inhibition of necroptosis through genetic (MLKL knockout) or pharmacological (RIPK3 inhibition) strategies, reverts age‐associated hippocampal changes, restores synaptic function, and reverses learning and memory to youthful levels.

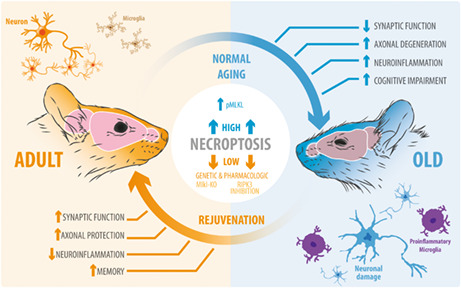

**Abbreviations**
AxD: axonal degneration
DG: dentate gyrus
fEPSP: field excitatory post‐synaptic potential
FJC: Fluoro Jade C
IL: interleukin
LTP: long‐term potentiation
MLKL: mixed lineage kinase domain‐like
Mlkl‐KO: Mlkl‐knockout mice
MS: mass spectrometry
MWM: Morris water maze
NF: neurofilament
non‐pNF: non‐phosphorylated neurofilament
RIPK3: receptor‐interacting kinase 3
SA‐βgal: SA‐βgalactosidase
TNF‐α: tumor necrosis factor alpha
WT: wild‐type

## 1 INTRODUCTION

The current rise in human life expectancy is not precisely accompanied by an equivalent increase in healthspan (Aburto et al., 2020), the functional and disease‐free period of life in the elderly (Hansen & Kennedy, 2016). The impact of age on brain function is unquestionable, being the main risk factor for the development of neurodegenerative diseases and cognitive disabilities (Agüero‐Torres et al., 2002; Duan et al., 2020; Wyss‐Coray, 2016).

As a fundamental structure for human cognition, the hippocampus is particularly vulnerable to the deleterious effects of aging (O'Shea et al., 2016; Wimmer et al., 2012). Microstructural changes at the synaptic level are correlated with learning and memory impairment during aging. Decreased number of axospinous synapses have been described in the aged hippocampus (Geinisman et al., 1992). The reduced number of synaptic contacts is correlated with a decreased presynaptic fiber potential due to a reduction of axons (Barnes & McNaughton, 1980), contributing to the impaired synaptic plasticity and cognitive deficits evidenced in aged organisms (Rosenzweig & Barnes, 2003). In addition, white matter abnormalities and axonal degeneration (AxD) have been identified in aged brains of diverse species (Peters et al., 2000; Stahon et al., 2016), particularly in the hippocampus, and correlated with impaired memory performance in humans (Radhakrishnan et al., 2020). Due to the importance of axonal integrity on hippocampal function and progression of cognitive decline in the elderly (Marner et al., 2003), it is imperative to determine the mechanism of AxD during aging. We have shown that necroptosis is involved in mechanical and chemical‐induced AxD (Arrázola et al., 2019; Hernández et al., 2018). Recently, in a model of neuronal inflammation it has been demonstrated that necroptosis activates Sarm1, a central executioner of pathological AxD (Gerdts et al., 2013; Ko et al., 2020; Osterloh et al., 2012). Necroptosis is an alternative form of programmed cell death triggered by the tumor necrosis factor under caspase‐8 inhibitory conditions and characterized by a necrotic‐like and pro‐inflammatory response (Holler et al., 2000; Seo et al., 2021). Receptor‐interacting kinase 1 (RIPK1) recruits and phosphorylates RIPK3, which in turn phosphorylates the mixed lineage kinase domain‐like protein (MLKL). MLKL oligomerizes and translocates to the plasma membrane, disrupting membrane integrity followed by the release of cellular components, an exacerbated inflammatory response, and cell death (Samson et al., 2020).

Age‐associated increase in low‐grade sterile inflammation contributes to the progression of age‐associated diseases (Franceschi & Campisi, 2014; Kennedy et al., 2014; López‐Otín et al., 2013). Recent studies have depicted the importance of necroptosis in the aging of the mouse male reproductive system (Li et al., 2017) and the epididymal white adipose tissue (Deepa et al., 2018). In the context of brain aging, several age‐related neurodegenerative conditions with prominent AxD and neuroinflammation as common features have shown increased necroptosis activation in the brain, associated with functional impairment (Caccamo et al., 2017; Iannielli et al., 2018; Ito et al., 2016; Oñate et al., 2020; Re et al., 2014; Salvadores et al., 2022). Necroptosis activation has been recently described in the cortex and hippocampus of aged mice, and correlated to age‐related neuroinflammation (Thadathil et al., 2021), and in the basolateral amygdala in a senescence‐accelerated mouse model of aging, and associated with reduced social interaction (Zhang et al., 2022). However, the involvement of necroptosis in the progression of normal brain aging and its impact on the age‐associated memory decline remain unexplored.

Here, we investigated the role of necroptosis in the progression of neurodegeneration in the hippocampus and its impact in brain function along aging. Necroptosis increased in the hippocampus of aged mice and accompanied with evident features of AxD. Loss of *Mlkl* was sufficient to delay age‐related AxD and neuroinflammation in the hippocampus; a youthful phenotype also displayed at the synaptic and functional level. Restored synaptic transmission and facilitation were accompanied by improved learning and memory in aged mice deficient for *Mlkl*. Short‐term inhibition of RIPK3 in aged mice demonstrated to be extraordinarily effective on reverting neurodegeneration and hippocampus‐dependent functional impairment. Finally, a label‐free quantitative proteomic analysis demonstrated that necroptosis inhibition improved the aged hippocampal proteomic profile, restoring the levels of key protein pathways associated with brain aging. Our study reveals that necroptosis contributes to the age‐associated deterioration of axons, affecting hippocampal neuronal connectivity and cognitive functions, such as learning and memory performance, of aged mice. Therefore, necroptosis inhibition constitutes a potential strategy for the development of therapeutic tools to treat age‐related brain disabilities and brain rejuvenation.

## 2 RESULTS

### 2.1 Necroptosis activation in the hippocampus of the aged mice

**FIGURE 1:**
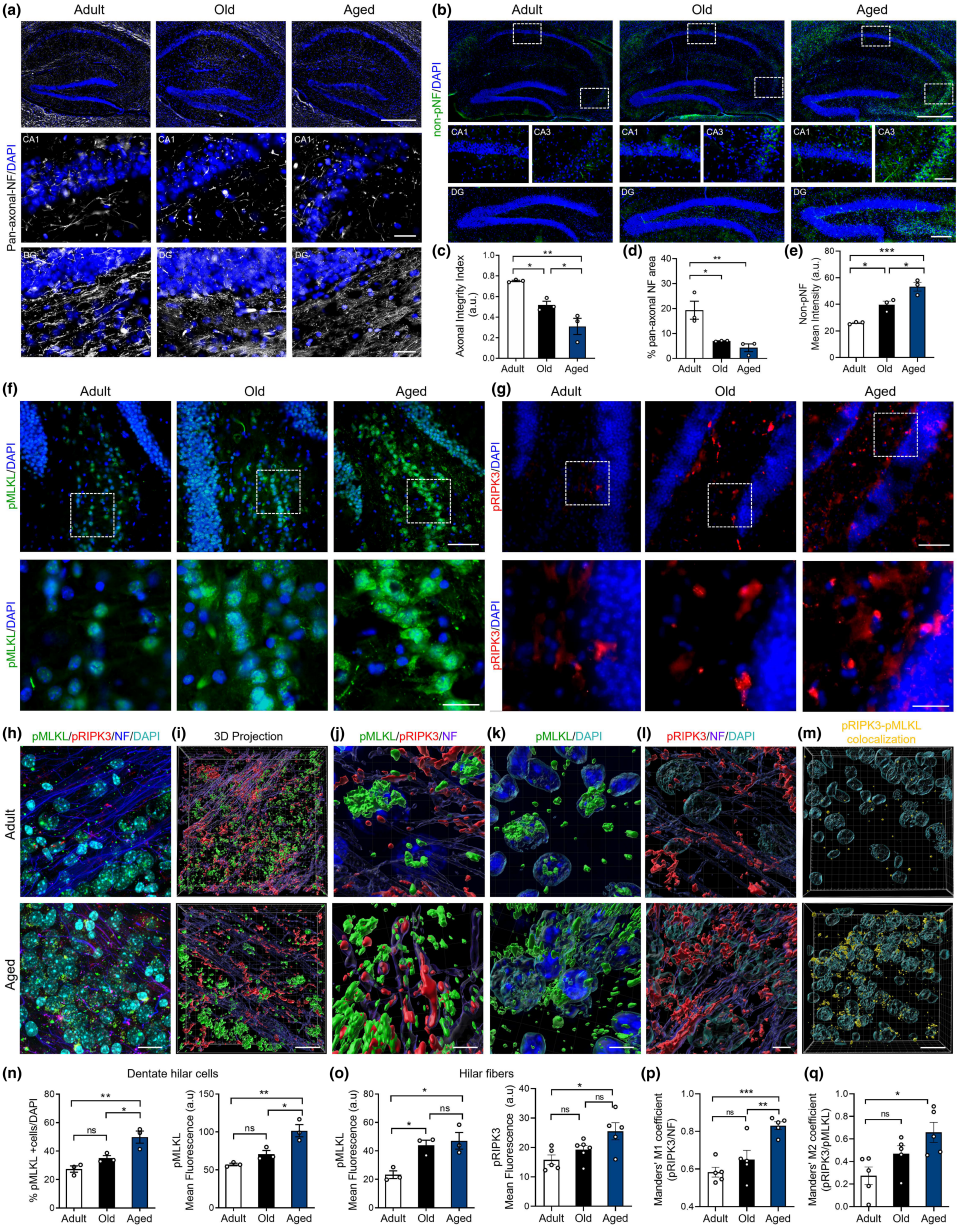
Progression of axonal degeneration coupled with necroptosis activation in the hippocampus during aging. Hippocampal sections of adult, old, and aged mice stained with (a) pan‐axonal NF (bars: 500 μm, hippocampus; 10 μm, crop), and (b) non‐phosphorylated neurofilament (non‐pNF) (bars: 500 μm, hippocampus; 200 μm, DG; 100 μm, CA1/CA3). DAPI, nuclei (blue). (c) Axonal integrity index and percentage of axonal area in the hippocampus (d). (e) Non‐pNF mean intensity in the hilus along aging. (f, g) MLKL and RIPK3 phosphorylation (pMLKL, green; pRIPK3, red; bars: 25 μm; 10 μm for magnification) (h, i) Maximal and 3D projection of confocal images, respectively, showing pMLKL (green), pRIPK3 (red), NF (blue), DAPI (cyan); bar: 15 μm. (j–l) magnified 3D images, bar = 5 μm. (m) 3D projection of the pMLKL/pRIPK3 colocalization channel (yellow), bar: 15 μm. (n) Percentage of pMLKL positive hilar cells normalized to the total cell number in the hilar area, and pMLKL mean intensity. (o) pMLKL and pRIPK3 mean intensity in hilar fibers along aging. (p) pRIPK3/NF Mander's colocalization analysis. (q) pRIPK3/pMLKL Mander's colocalization analysis. Values, mean ± SEM, are the result of the analysis of *n* = 3–6 mice per group. One‐way ANOVA with Tukey analysis for multiple comparisons, *p < 0.05; **p < 0.01; ***p < 0.005.

To establish the progression of hippocampal degeneration through aging, we used adult (3–6 months), old (12–15 months), and aged (more than 20 months) mice. Neurodegeneration evaluated by Fluoro Jade C (FJC) staining significantly increases in the hippocampus of aged mice (Figure S1). FJC staining also increased along aging in other brain regions, including the striatum, cerebellum, and spinal cord (Figure S1). Neuronal death or apoptosis, evaluated by caspase‐3 activation and TUNEL analysis, were not significantly detected in the hippocampus of any of the age groups studied (Figure S2). Considering that healthy aging is a process affecting predominantly the white matter in the brain (Piguet et al., 2009; Radhakrishnan et al., 2020), we evaluated age‐associated AxD in the hippocampus, by studying the expression of two phosphorylated forms of NFs: highly phosphorylated NFs which are predominant in stable axons, and non‐phosphorylated NF (non‐pNF) immunoreactivity, associated with axonal damage (Nadeem et al., 2016; Petzold, 2005). Axonal integrity was analyzed by calculating the Axonal Integrity Index (Figure S3) from pan‐axonal NF‐stained axons (stable axonal NFs) in the hippocampus (Figure 1a). The integrity and percentage of NF‐positive axons decreased in old and aged hippocampus compared with adult mice (Figure 1a, c, d). Almost undetectable immunoreactivity of non‐pNF was observed in the hippocampus of adult mice, while a progressive increase was evidenced during aging in different subfields of the hippocampus (Figure 1b, e).

We recently demonstrated that mechanical and chemical‐induced axonal degeneration is regulated by necroptosis (Arrázola et al., 2019). To determine whether necroptosis is involved in age‐associated AxD, we assessed necroptosis activation by the expression of the phosphorylated forms of MLKL (pMLKL) and RIPK3 (pRIPK3) in different hippocampal subfields, including hilar axons of the DG (Figure 1f–q) and the CA3‐CA1 projecting Schaffer collateral axons (Figure S4). The number of pMLKL positive hilar cells and pMLKL mean intensity increased in aged mice, as did pRIPK3 (Figure 1f, g). Axonal pMLKL and pRIPK3 staining was evaluated by confocal microscopy in pan‐axonal‐NF positive hilar fibers of the DG (Figure 1h). Interestingly, pMLKL mean intensity increased earlier in DG axons compared with dentate hilar somas throughout aging, reaching significant differences in the old mice group (Figure 1o). Phospho‐RIPK3 mean intensity also increased along aging in the hilus (Figure 1o). Three‐dimensional projection of confocal images also showed increased pMLKL and pRIPK3 levels in the hilus (Figure 1h–j). The increase in pMLKL levels in the hilus was also accompanied by changes in the pattern of pMLKL signal, from almost non‐detected in adult mice axons, diffuse in the old group, to finally become punctuated in axons of aged mice (Figure 1i, j and Figure S4). These pMLKL aggregates have been associated with MLKL oligomerization and its translocation to the plasma membrane, two key steps for necroptosis execution (Samson et al., 2020). A similar pMLKL pattern was observed by immunohistochemistry against pMLKL in DG hilar cells and in Schaffer collateral axons of the CA3‐CA1 circuit of aged mice (Figure S4). Interestingly, necroptosis activation was also evident by an age‐dependent translocation of pMLKL from the nucleus to the cytoplasm (magnified Figure 1f, k), as previously described in vitro (Yoon et al., 2016). Moreover, the colocalization analysis showed increased overlapping of pRIPK3 within axons (Figure 1l, p), but also between pRIPK3 and pMLKL (Figure 1m, q), altogether confirming necroptosis activation in the hippocampus of aged mice. Increased pMLKL signal was also observed in other brain regions with defined axonal subfields, as axonal tracts in the striatum, the cerebellar white matter and the ventral horn of the spinal cord, which also showed progressive AxD along aging (Figure S5). Only at later stages of life, necroptosis markers were associated with a low and non‐significant percentage of cell death (Figure S2). These results indicate that necroptosis is activated early during aging in the hippocampus, a brain region that is considered one of the most vulnerable to the detrimental effects of aging, affecting learning and memory (Spiegel et al., 2013).

### 2.2 Age‐induced axonal degeneration and neuroinflammation are reduced in MLKL knockout mice

**FIGURE 2:**
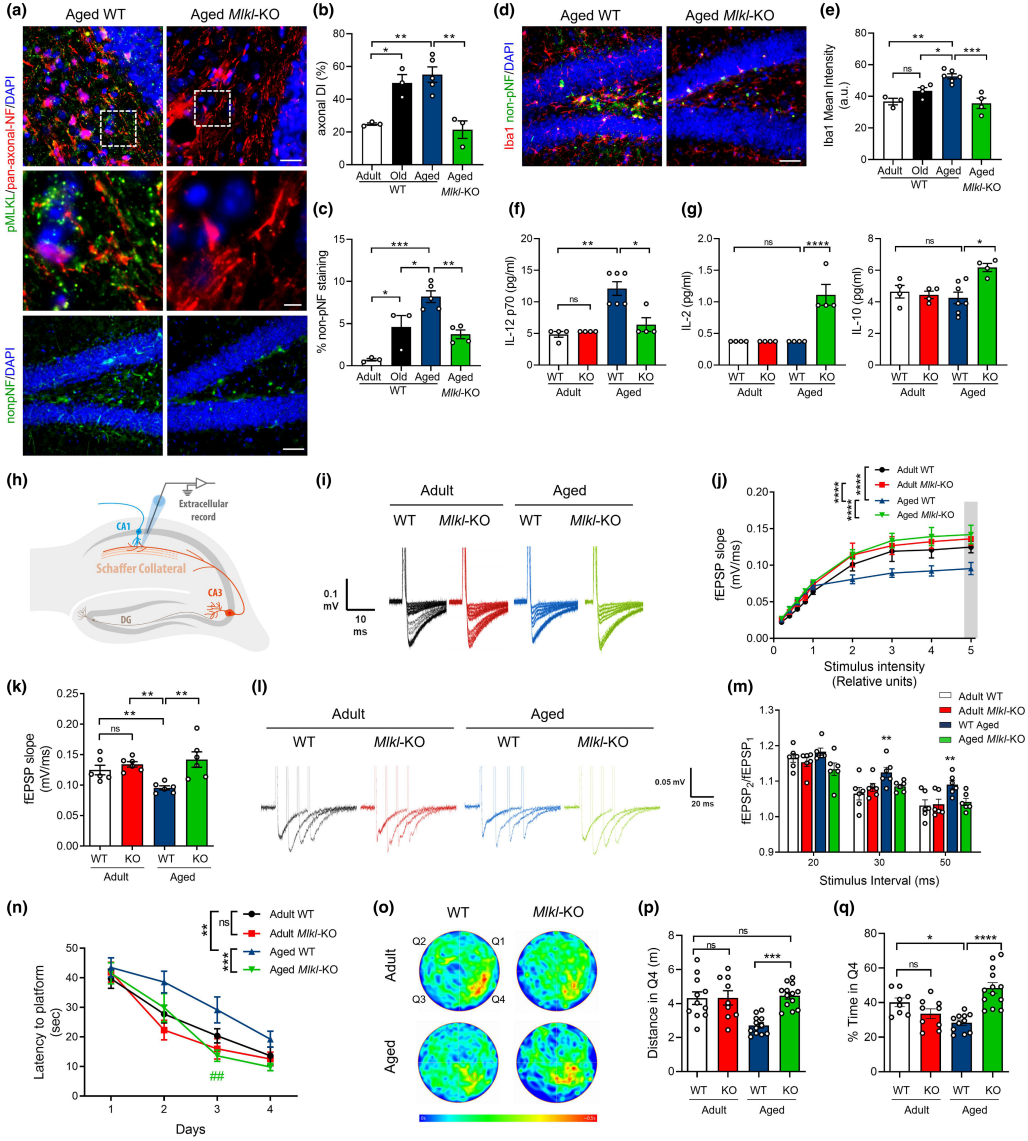
*Mlkl* deficiency protects from neurodegenerative hallmarks and improves hippocampal function and memory in aged mice. Axonal degeneration and neuroinflammation in the hippocampus of aged WT and *Mlkl*‐KO mice compared with the normal aging curve (*N* = 3–5 mice per group). (a, b) Axonal degeneration index (DI) calculated from pan‐axonal‐NF images (red, bars: 25 μm; 5 μm, insets). (a, c) Signs of neurodegeneration by non‐pNF staining (bar: 50 μm), plotted as percentage of non‐pNF area. (d, e) Microglia activation quantified as Iba1 mean intensity (bar: 50 μm). (f, g) Cytokines levels in the hippocampus of adult vs aged WT and *Mlkl*‐KO. Absolute cytokine levels (pg/mL) of IL‐12, IL‐2, and IL‐10 (N = 4–7 mice per group). (h) Field excitatory post‐synaptic potential (fEPSP) measured in hippocampal slices in the CA3‐CA1 circuit. (i, j) Average traces of the evoked potentials plotted as fEPSP slope upon several stimulus intensity. (k) Average fEPSP slope at the highest stimulus (gray bar in j). (l, m) Paired pulse facilitation as facilitation index (fEPSP_2_/fEPSP_1_), *n* = 6 slices per group. (n) Morris water maze (MWM) navigation task. Escape latency measured as the time to reach the hidden platform during the learning task in adult WT (*n* = 11), adult *Mlkl*‐KO (*n* = 10), aged WT (*n* = 13) and aged *Mlkl*‐KO mice (*n* = 13). (o) Mean heatmaps (pseudo‐color) specify location of each mice cohort along time during memory testing (day 5). Q4 quadrant, initial location of the hidden platform during training. (p, q) Travelled distance and time spent in Q4 during memory assessment at day 5. Values, mean ± SEM, *p < 0.05; **p < 0.01; ***p < 0.005; ****p < 0.001. One‐way ANOVA with Tukey analysis for multiple comparisons. RM‐ANOVA plus Sidak's multiple comparison test for the learning curve data analysis. ***p = 0.0005, significance between aged WT and *Mlkl*‐KO curves; **p = 0.0097, differences between adult and aged WT mice; ^##^ *p* = 0.0035, day 3 significance between aged WT vs *Mlkl*‐KO.

Due to the indispensable role of MLKL in executing necroptosis, we evaluated whether age‐dependent AxD in the hippocampus was modified in aged *Mlkl*‐knockout mice (*Mlkl*‐KO) (Wu et al., 2013). Interestingly, AxD in pan‐axonal NF‐stained axons was significantly reduced in aged *Mlkl*‐KO mice at levels comparable with adult mice (Figure 2a, b). Moreover, non‐pNF degenerated axons profusely present in aged WT hippocampus were almost undetected in aged *Mlkl*‐KO mice, at levels equivalent to younger WT mice (Figure 2a, c). As expected, we observed age‐dependent AxD coupled with neuroinflammation (Hwang et al., 2018) (Figure S6). The increased number of microglia in the hippocampus of aged mice was prevented in aged *Mlkl*‐KO mice, as it was the microglia overlapping with degenerating axons (Figure S7), as previously described (Thadathil et al., 2021). Accordingly, measurement of Iba1 mean intensity also indicated that aged *Mlkl*‐KO mice present less microglia activation than their WT littermates (Figure 2d, e). To confirm the contribution of necroptosis to the inflammatory state of the aging brain, we measured the levels of several cytokines and chemokines in hippocampal lysates and serum of adult versus aged WT and *Mlkl*‐KO mice by Luminex High Performance Assay (Table S1). Three of the 12 cytokines analyzed showed significant changes under *Mlkl* deficiency in the hippocampus of aged mice. The levels of the pro‐inflammatory cytokine IL‐12 decreased in aged *Mlkl*‐KO hippocampus compared with aged WT mice, reaching levels comparable with adult WT mice (Figure 2f). Interestingly, the anti‐inflammatory cytokines IL‐2 and IL‐10 significantly increased in the hippocampus of aged *Mlkl*‐KO mice (Figure 2g). Moreover, the systemic pro‐inflammatory profile also decreased in serum samples from aged *Mlkl*‐KO mice (Figure S8). These results indicate that necroptosis contributes to brain inflammation by modulating both pro‐ and anti‐inflammatory cytokines.

### 2.3 Loss of MLKL improves hippocampal synaptic transmission, learning, and memory in aged mice

To further study whether necroptosis activation affects hippocampal function along aging, we first performed electrophysiological recordings of the CA3‐CA1 synapses to evaluate synaptic transmission. Extracellular field‐excitatory post‐synaptic potentials (fEPSP) were registered in hippocampal slices (Figure 2h). An age‐dependent decrease in fEPSP slope was observed in WT mice. Nevertheless, in aged *Mlkl*‐KO mice, the fEPSP slope was maintained at levels comparable to adult WT mice (Figure 2i–k). To specifically evaluate whether axonal alterations contribute to an age‐dependent decrease in synaptic transmission, we analyzed the facilitation index (fEPSP_2_/fEPSP_1_) using a paired‐pulse stimulation protocol (Figure 2l). The increased facilitation index observed in WT aged mice indicates a decreased neurotransmitter release probability from the axonal compartment (pre‐synapse). Interestingly, the facilitation index of aged *Mlkl*‐KO mice was comparable to adult WT mice (Figure 2m). These results indicate that *Mlkl* deficiency delays the loss of synaptic strength in the hippocampus inherent to brain aging, mainly preventing axonal function defects in aged mice.

Since memory capabilities depend on proper hippocampal function and both are affected by age (Burke & Barnes, 2006; Yang et al., 2019), we evaluated whether *Mlkl* loss improves spatial learning and memory in aged mice using the Morris water maze (MWM) navigation task. The learning curve of aged *Mlkl*‐KO mice was significantly faster than those of aged WT animals and reached a reduced latency to find the platform, showing comparable escape time to adult animals (Figure 2n). No differences were observed between adult WT and *Mlkl*‐KO mice in the learning curve. To evaluate memory, mice were challenged 24 h after training to find the original location of the platform. Aged *Mlkl*‐KO mice travelled a larger distance and spent more time exploring in the target quadrant Q4 compared to aged WT animals (Figure 2p, q), without significant changes in the mean swimming speed between both groups (Figure S9), demonstrating that loss of *Mlkl* prevents learning and memory loss associated with aging.

Altogether, these results demonstrate that an age‐associated increase in brain necroptosis induces degeneration of axons in the hippocampus, thereby depressing synaptic transmission, and impairing hippocampal‐dependent functions, such as learning and memory in aged mice.

### 2.4 Pharmacological inhibition of necroptosis reverts key signs of brain aging, improving hippocampal function and memory

**FIGURE 3:**
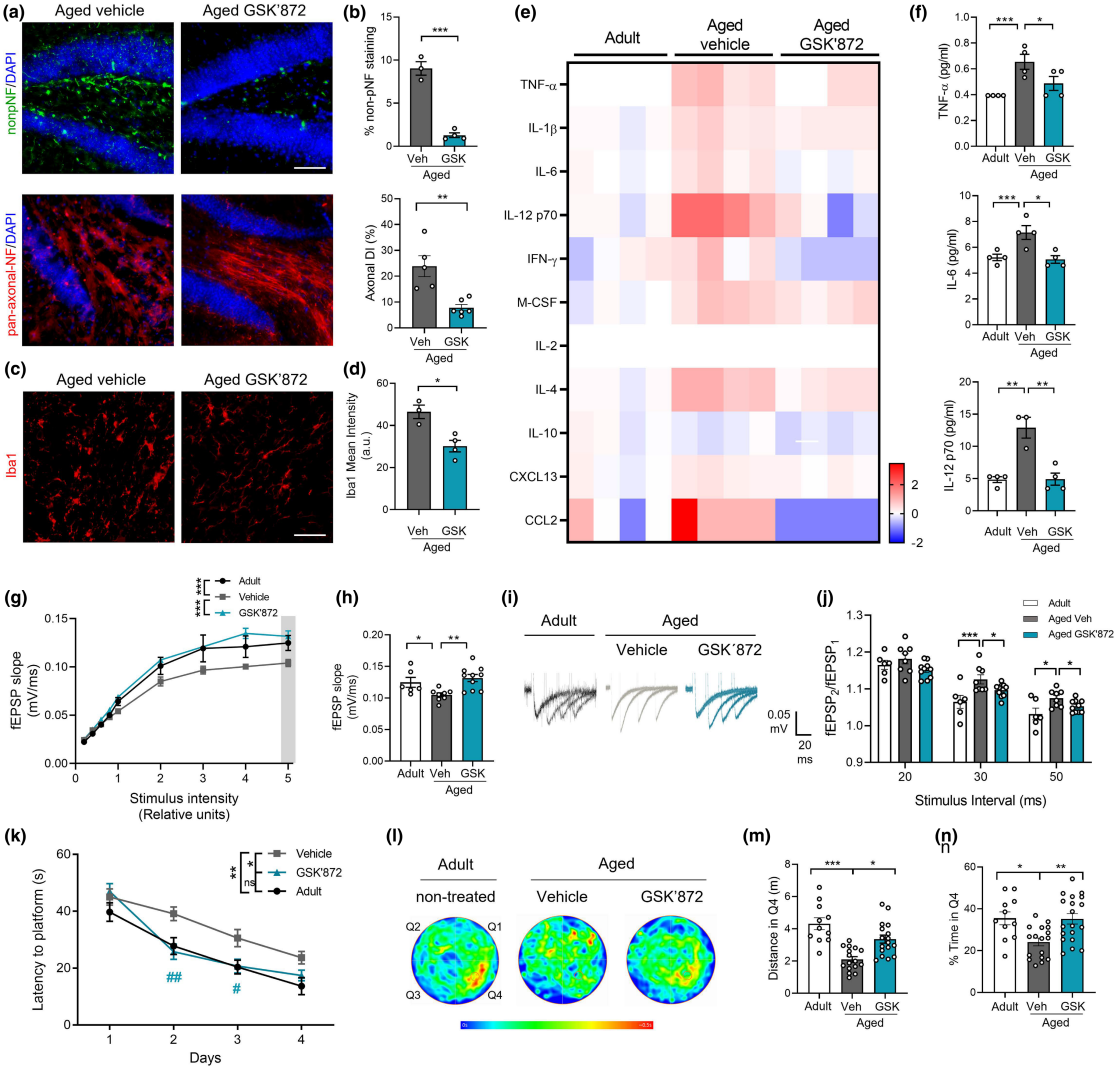
Short‐term inhibition of necroptosis reverts hippocampal degeneration restoring electrophysiological function and memory in aged mice. Aged mice treated with vehicle or GSK’872. (a) Non‐pNF (green) and pan‐axonal NF (red) staining (bar: 50 μm). (b) Percentage of non‐pNF stained area (top) and axonal degeneration index (bottom) of aged vehicle and GSK’872‐treated mice (*n* = 3–6 mice per group). (c, d) Microgliosis (bar: 50 μm) measured as Iba1 mean intensity. (e) Heatmap, fold change levels of cytokines analyzed in the hippocampus (*n* = 4, separated in each column). (f) Absolute levels (pg/mL) of TNF‐α, IL‐6 and IL‐12p70. (g) Average traces of the evoked potentials recorded and used to plot the fEPSP slope upon several stimulus intensity. (h) Average fEPSP slope from the highest stimulus (gray bar in g). (i, j) Facilitation index (fEPSP_2_/fEPSP_1_), *n* = 6 slices per group. Values, mean ± SEM, *p < 0.05; **p < 0.01; ***p < 0.005, one‐way ANOVA with Tukey analysis for multiple comparisons. (k) MWM test, escape latency of adult (*n* = 11), aged vehicle (*n* = 17), and aged GSK’872‐treated mice (*n* = 19). (l) Swimming heatmaps (pseudo‐color) show average location along time during memory testing (Day 5). (m, n) Travelled distance and the time spent in Q4 during memory assessment at Day 5 were analyzed by one‐way ANOVA with Tukey analysis for multiple comparisons, *p < 0.05; **p < 0.01; ***p < 0.005. Learning curve data were analyzed by RM‐ANOVA with Sidak's multiple comparison test: *p = 0.0191, significance between vehicle and GSK’872 curves; **p = 0.0019, differences between adult and vehicle groups. Differences between vehicle and GSK’872 groups at Days 2 and 3 correspond to ^##^ *p* = 0.0022 and ^#^ *p* = 0.04238, respectively. Non‐significant differences were observed between aged GSK´872 and adult curves.

To further explore the role of necroptosis in brain aging, we use the RIPK3 inhibitor, GSK’872 (Salvadores & Court, 2020; Yang et al., 2017). Diffusion pumps were filled with vehicle or GSK’872, and intraperitoneally implanted to continuously diffuse the inhibitor in 23‐month‐old mice for 28 days (2 mg/kg GSK’872 at 0.11 μL/h). Pharmacokinetic studies shown measurable levels of GSK’872 1 h post‐administration in the brain (187.6 ± 17.11 nm) and plasma (18.32 ± 0.85 μm) in aged animals after a single i.p. dose of 10 mg/kg. Importantly, GSK’872 treatment leads to decreased pRIPK3 signal in the hilus of aged GSK’872‐treated mice (Figure S10). Remarkably, aged GSK’872‐treated mice showed decreased non‐pNF staining and reduced AxD index in comparison with vehicle‐treated mice of the same age (Figure 3a, b). Moreover, decreased microglia activation was also observed, indicating that a short‐term treatment with GSK’872 is capable of reverting one of the main signs of brain inflammation associated with aging (Figure 3c, d).

Since RIPK3 is involved in pathways that regulate cytokines secretion (Orozco & Oberst, 2017), we performed Luminex High Performance Assay to detect changes in a pool of selected cytokines (Table S1). Interestingly, the hippocampus of GSK’872 treated mice showed a pattern of cytokine levels highly similar to those observed in untreated adult mice (Figure 3e). The analysis reveals that RIPK3 inhibition mainly reduces pro‐inflammatory cytokines, such as TNF‐α, IL‐6, and IL‐12, reaching youthful‐like cytokine levels equivalent to adult mice (Figure 3f). A similar profile of decrease in pro‐inflammatory cytokines was observed systemically in serum samples of GSK’872 treated mice (Figure S8).

At the functional level, electrophysiological recordings in the hippocampus demonstrated equivalent fEPSP between aged mice treated with GSK’872 and untreated adult mice. Average traces showed significant differences between aged vehicle‐treated mice versus those that received the RIPK3 inhibitor (Figure 3g, h). In addition, facilitation index was significantly lower in the hippocampus of aged mice treated with GSK’872 compared with the aged vehicle mice (Figure 3i, j), showing that late and short‐term necroptosis inhibition can revert the loss of synaptic strength in aged mice. Remarkably, RIPK3 inhibition significantly improved learning in aged mice (Figure 3k). Memory assessment indicated that aged mice with GSK’872 treatment spent more time in the target quadrant and travelled larger distance in Q4 compared with aged vehicle‐treated mice, at levels equivalent to adult untreated mice (Figure 3l–n). Thus, RIPK3 inhibition is capable to recover aged mice from learning and memory impairment.

These results demonstrate that a short‐term systemic administration of GSK’872, in a late phase of the lifespan of mice, can revert key hallmarks of brain aging, including AxD, neuroinflammation, and age‐associated memory impairment, proposing the inhibition of necroptosis as an attractive therapeutic target to improve memory in the elderly.

### 2.5 Proteomic analysis in the hippocampus of aged mice with inhibition of necroptosis reveals improvement in key hallmarks of aging and brain rejuvenation

In order to elucidate the specific molecular alterations underpinning our necroptosis‐inhibitory approaches toward reducing aging phenotypes, we employed a state‐of‐the‐art single‐shot, label‐free quantitative proteomic approach. Hippocampus of adult and aged WT mice, aged *Mlkl*‐KO mice, and aged GSK’872‐treated mice were subjected to single‐shot label‐free mass spectrometry (see workflow in Figure S11a), obtaining a high degree of coverage of the proteome with almost 7000 proteins detected (Figure S11b).

**FIGURE 4:**
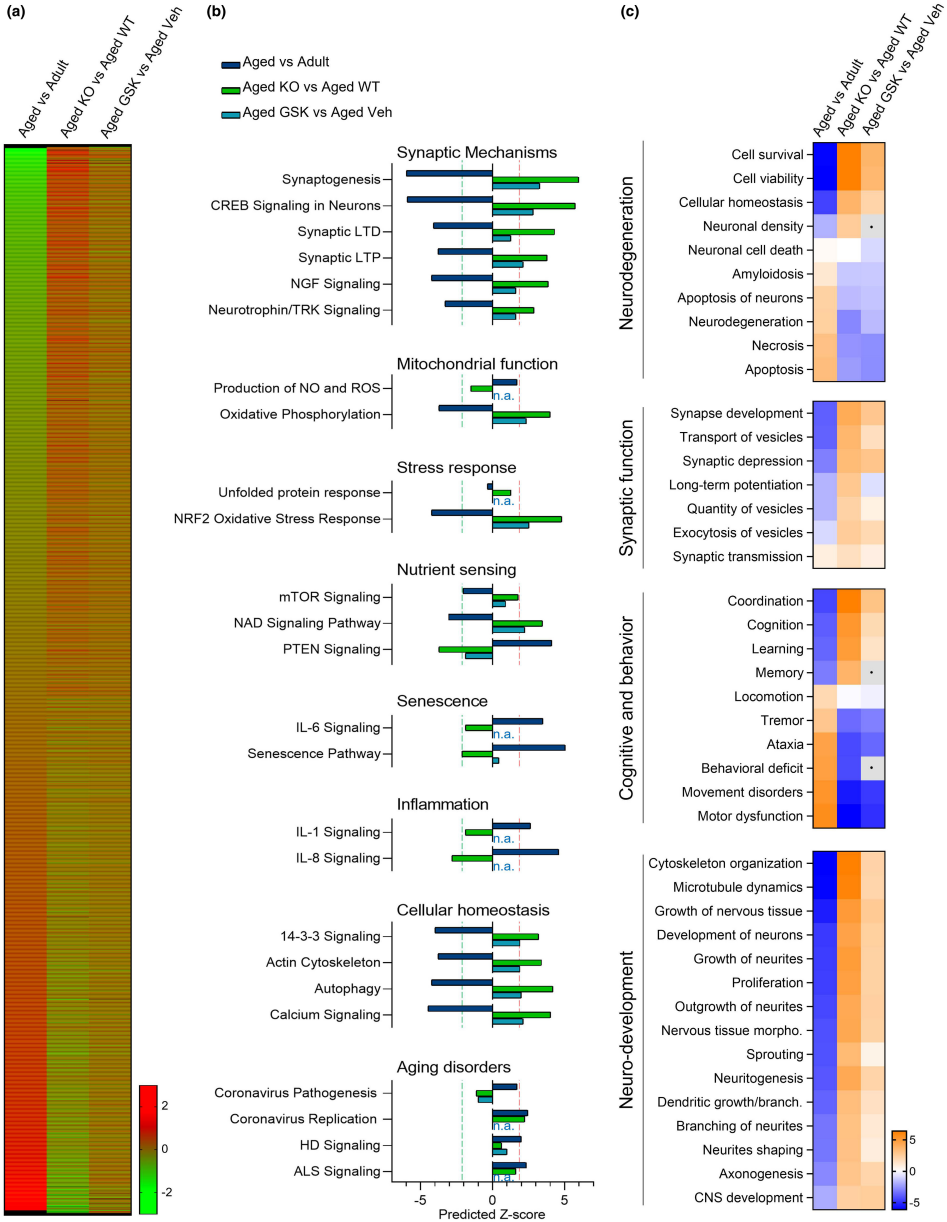
Proteomic analysis reveals opposing directionality in protein expression and aging‐associated biofunctions between normal aging and necroptosis‐targeted inhibition. (a) Heat map, protein expression profile between “normal” aging (aged vs adult) and necroptosis‐inhibited processes in aged mice (*Mlkl*‐KO and GSK’872). Mean expression ratio (log 2) of *n* = 4 mice per experimental group vs their respective controls. Upregulation (red) and downregulation (green). (b) Canonical pathways classified as aging hallmarks. A predicted *z*‐score >2 or < −2 (indicated by dotted lines) is considered significant when using IPA. (c) Diseases and biofunctions implicated in brain rejuvenation. Heatmaps, predicted activation (orange), and predicted inhibition (blue) scores between “normal” aging, and both *Mlkl‐*KO or GSK’872‐treated mice.

After relative expression ratios were calculated and expression profile clustering were performed, we identified subsets of proteins exhibiting opposing directionality in expression between the “normal” aging proteome (aged vs adult animals) and the proteomes of animals subjected to genetic (aged *Mlkl*‐KO vs aged WT) or pharmacological (aged GSK’872 vs aged vehicle) inhibition of necroptosis (Figures S11 and S12). In doing so, it was possible to reduce the number of correlative candidate proteins to 2516 proteins whose expression increases and 2307 which decrease in the aging hippocampus and where genetic and pharmacological modulation leads to a degree of reversion (Figure 4a). Pathway analysis confirmed opposing directionality in the activation status of numerous biological processes between “normal” and necroptosis‐targeted aged animals. These include several canonical pathways and biological function annotations previously implicated in normal aging (Kennedy et al., 2014; López‐Otín et al., 2013) (see Figure S13a) and in brain rejuvenation (Bouchard & Villeda, 2015; Wyss‐Coray, 2016) (Figure 4b and Figure S13b). Most of the molecular cascades belonging to these pathways are typically associated with neurodegeneration and/or neuronal aging, including molecular cascades involved in *synaptic mechanisms*, *senescence*, and *cellular homeostasis* (Figure 4b).

In order to explore the contribution of necroptosis in age‐associated neuronal dysfunction, we evaluated several biological functions affected by normal aging in our proteomic analysis. These biofunctions were classified considering their contribution in central brain functions, designated as *neurodegeneration*, *synaptic function*, *cognitive and behavior*, and *neuronal development*. It is interesting to note that some of the patterns of change of these biofunctions show opposing directionality in both the genetic and pharmacological approaches of necroptosis inhibition compared with normal aging (Figure 4c). Remarkably, key molecular and cellular functions associated with neurodegeneration, neuronal integrity and function, showed a clear reversion in the context of pathway analysis in necroptosis‐targeted aging in comparison with normal aging (Figure 4c). This analysis demonstrated that the inhibition of necroptosis supports proper brain function in aged animals, improving some key hallmarks of aging and restoring (in the case of GSK’872) relevant functions involved in brain rejuvenation.

### 2.6 Necroptosis inhibition induces synaptic long‐term potentiation in aged mice

**FIGURE 5:**
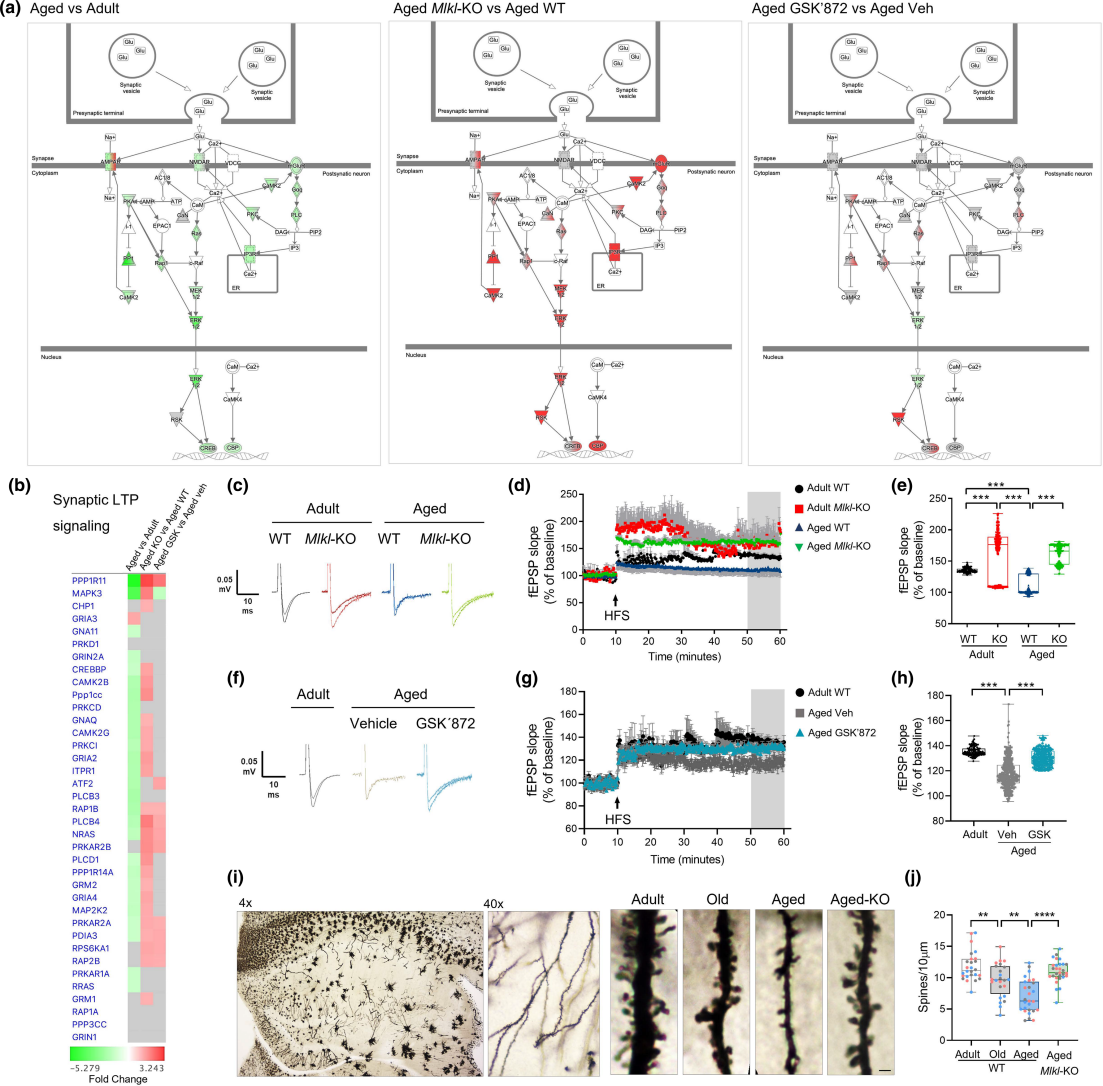
Increased synaptic long‐term potentiation in the necroptosis‐inhibited aging process. (a) Canonical pathway annotation of “Synaptic long‐term potentiation signaling”. Upregulation (red) and downregulation (green) compared to control. Molecules in grey fell below the 20% cutoff. Molecules in white were not present within input dataset but changed less than 20% in analysis. Solid connecting lines: direct interaction; dashed connecting lines: indirect interaction. (b) Heat map of individual proteins assigned to the “Synaptic long‐term potentiation signaling”. Changes were expressed as fold change. (c–e) Synaptic plasticity measured as LTP magnitude in the CA1‐CA3 hippocampal transmission of WT and *Mlkl*‐KO mice from adult and aged groups after high‐frequency stimulation (HFS). fEPSP slope plotted as individual values considering the last 10 min of recording. (f–h) LTP magnitude of aged GSK’872‐treated mice vs vehicle, registered for 1 h. Boxplots of the last 10 min of fEPSP slope. *N* = 4 mice per group (*n* = 8–10 slices per mice). (i, j) Dendritic spines visualized by Golgi‐Cox staining in the hippocampus along aging and compared with aged *Mlkl*‐KO mice (bar: 1 μm). Spines number normalized to 10 μm of dendrite length. (*n* = 3 mice per group; circles correspond to each dendrite measured per *n*). Values, mean ± SEM, **p < 0.01, ***p < 0.005, ****p < 0.001. One‐way ANOVA.

Among the cascades elucidated in our proteomic analysis, those classified as *synaptic mechanisms* showed the highest predicted z‐score (Figure 4b). Synaptic long‐term potentiation (LTP) is a key process directly related to learning and memory, and early impaired during aging (Lynch et al., 2006). The contribution of the synaptic LTP signaling at the level of individual molecules is visually illustrated in Figure 5a. These molecular changes are mitigated in the aging process of the *Mlkl*‐KO mice (red molecules) and partially reverted after necroptosis inhibition with GSK’872 in aged animals (Figure 5b). We therefore analyzed hippocampal synaptic plasticity by studying LTP magnitude in the CA3‐CA1 transmission. Using a high‐frequency stimulation protocol, we found that LTP induction was compromised in aged WT mice when compared to adult WT mice (Figure 5c–e). Surprisingly, adult *Mlkl*‐KO mice presented a higher LTP magnitude than the control WT group and the loss of *Mlkl* in aged mice restored LTP induction and maintenance beyond adult WT mice potentiation. Similar to KO experiments, we detected a reduction in LTP magnitude when adult WT mice were compared with aged vehicle‐treated mice. Surprisingly, only 1 month of GSK’872 treatment in aged mice was capable to improve LTP magnitude, reaching adult‐like levels (Figure 5f–h). As a correlate of learning and memory improvement, hippocampal long‐term synaptic plasticity also impacts dendritic spine remodeling (Engert & Bonhoeffer, 1999). We observed that the inhibition of necroptosis in aged *Mlkl*‐KO mice protects from the loss of spines in CA1 neurons of the hippocampus along aging (Figure 5i, j). Altogether, these results demonstrate that inhibition of necroptosis either by genetic knockout of *Mlkl* or by pharmacologic RIPK3 inhibition improved or restore synaptic plasticity in aged mice (functionally and morphologically), a synaptic process that is crucial to support brain rejuvenation (Wyss‐Coray, 2016).

### 2.7 Necroptosis activation contributes to the acquisition of the age‐associated senescent phenotype in the hippocampus during aging

**FIGURE 6:**
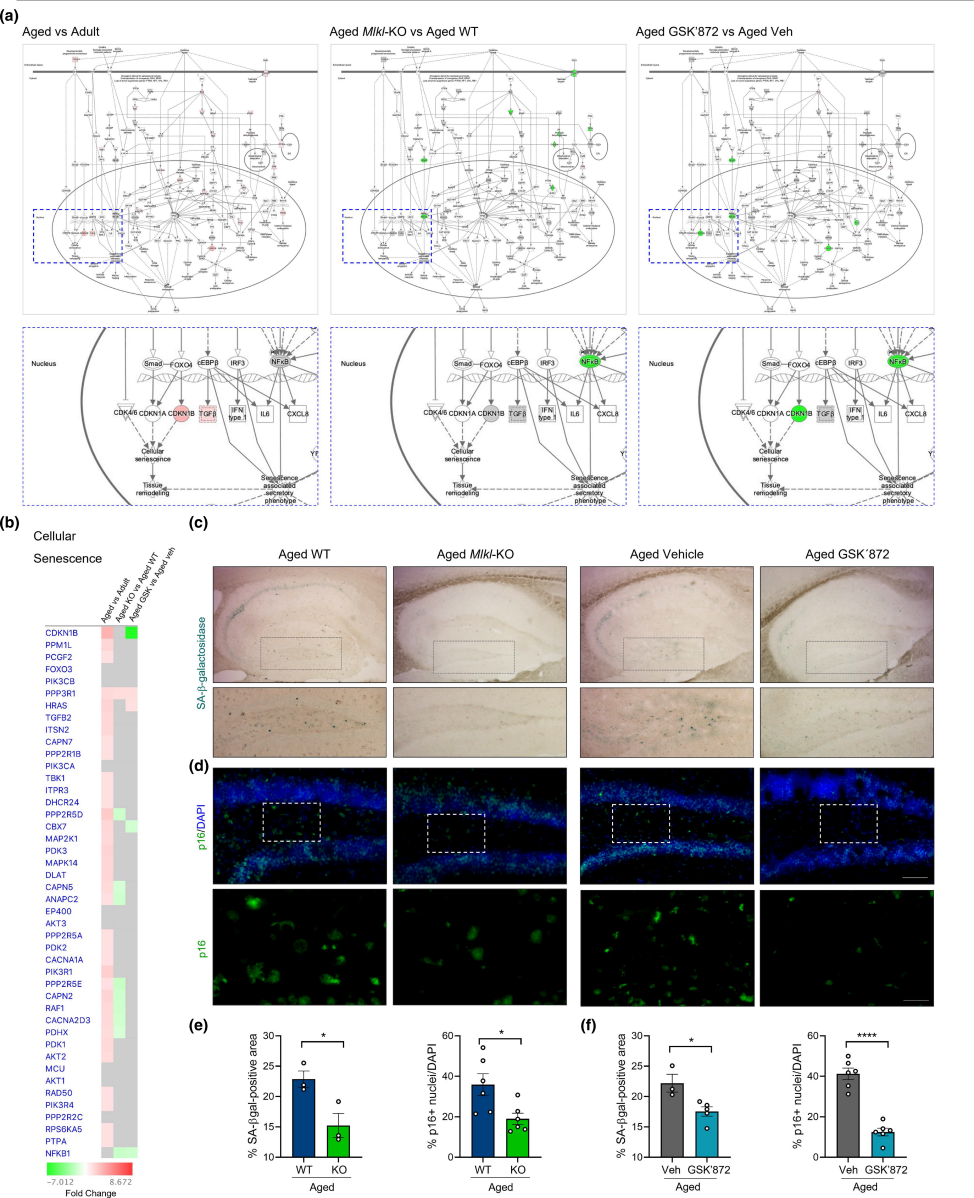
Decreased senescent phenotype in the hippocampus of aged mice with inhibited necroptosis (a) Canonical pathway annotation of “cellular senescence signaling”. Upregulation (red) and downregulation (green) compared to control. Molecules in grey fell below the 20% cutoff. Molecules in white were not present within input dataset but changed less than 20%. Solid connecting lines: direct interaction; dashed connecting lines: indirect interaction. Magnifications show target molecules involved in cellular senescence execution (blue dotted rectangle) (b) Heat map of individual proteins assigned to the “cellular senescence” annotation. Changes were expressed as fold change. (c) SA‐*β*galactosidase (SA‐*β*gal) activity (light blue) and (d) p16 levels (green) were quantified in the DG of aged WT, aged *Mlkl*‐KO, aged vehicle and aged GSK’872 treated mice (bar: 100 μm). Dotted boxes, magnifications of p16 positive cells in the hilus (bar: 30 μm). (e, f) Percentage of the SA‐*β*gal‐positive area and p16 positive nuclei (normalized against dapi) analyzed in the DG hilus of aged WT and Mlkl‐KO mice, and vehicle vs GSK’872‐treated mice, respectively. Values, mean ± SEM (*n* = 4–6 mice). Unpaired *T*‐test Student, *p < 0.05 and ****p < 0.001.

The accumulation of senescent cells in aged tissue, including the brain, is one of the most common features of aging (Jurk et al., 2012; Wang et al., 2009). As neuronal senescence has also been detected in the hippocampus of aged mice (Gorostieta‐Salas et al., 2021), we aimed to determine whether necroptosis contributes to the establishment of the senescent phenotype of hippocampal neurons along aging. From our proteomic data, the molecular changes associated with senescence were mostly prevented in the aging process of *Mlkl*‐KO mice and partially reverted after pharmacologic inhibition of necroptosis with GSK’872 in aged animals (Figure 6a). Interestingly, the expression profile of two key proteins involved in the acquisition of the senescent phenotype, namely CDKN1B and NFκB1 (Pruitt et al., 2013), were mainly reverted in both *Mlkl*‐KO and GK’872‐treated mice during aging (blue dotted insets, Figure 6a, b). We therefore evaluated reliable markers of cell senescence in the brain, such as SA‐βgalactosidase (SA‐βgal) activity and p16(INK4) levels (Cheng et al., 2017; Gorostieta‐Salas et al., 2021), measured in different subfields of the hippocampus of aged *Mlkl*‐KO and GSK’872‐treated mice (Figure S14 and Figure 6c, d). A significant reduction in SA‐βgal and p16 levels was observed in the hippocampus of aged *Mlkl*‐KO (Figure 6e) and GSK’872‐treated mice compared with their respective controls (Figure 6f), reinforcing the data obtained from the proteomic analysis, which overall indicates that age‐related activation of necroptosis contributes to the development of key pathological changes that are involved in brain aging.

## 3 DISCUSSION

Our findings reveal the involvement of necroptosis in the progression of AxD in the hippocampus during normal aging, uncovering relevant implications of necroptosis activation in age‐associated cognitive impairment, which might undermine healthy aging. Of note, most detrimental consequences of brain aging, including neuroinflammation, synaptic failure, and hippocampal‐dependent behavioral impairment, were prevented through inhibition of necroptosis by genetic or pharmacological means. In our mouse model of aging, neurodegeneration appears to be restricted to axonal‐enriched brain regions, such as the corpus striatum, the cerebellum, and white matter regions of the spinal cord (Figure S1). Corresponding with this observation, neurodegeneration was also noted in hippocampal subfields recognized as the richest axonal projection regions, including DG‐CA3‐CA1 axonal pathways (Ropireddy et al., 2011). The DG supports hippocampal neuronal connectivity by receiving entorhinal cortex information, which is transmitted to the CA3‐CA1 neurons to complete the hippocampal circuitry. DG neurons, particularly mossy cells of the hilus, are extremely vulnerable to degenerative insults (Santhakumar et al., 2000; Scharfman, 2016), and aging (West, 1993), compromising DG connectivity and increasing susceptibility to memory deficits in aged individuals (Amani et al., 2021; Dillon et al., 2017). This evidence together with the fact that hippocampal white matter abnormalities in human brains are associated with age‐dependent cognitive deterioration (Radhakrishnan et al., 2020) is tightly aligned with our results showing increased necroptosis in hilar cells, and an early activation in DG hilar axons along aging (Figure 1 and Figure S4). Furthermore, we observed that necroptosis activation in the hippocampus during the aging process occurs before the appearance of the first signs of cell death, which is evident only at late stages of life (Figure S2c). These observations suggest that the activation of necroptosis in DG hilar cells and axons may contribute to the vulnerability of this area to degenerate along aging, to adversely impact synaptic function and hippocampal‐dependent behavioral performance. The functional role of necroptosis as a crucial pathogenic determinant in experimental models of human diseases has been of increasing interest in the field (Choi et al., 2019). Increased pMLKL levels in aged mice suggest that necroptosis could be also considered as a biomarker of aging progression. Of note, aged *Mlkl*‐KO mice presented a youthful phenotype in the hippocampus, reaching levels of AxD comparable with those observed in younger mice (Figure 2).

As a chronic inflammatory condition, aging also influences the inflammatory status of the brain (Kennedy et al., 2014), mainly through microglia activation and increase of pro‐inflammatory cytokines (Barrientos et al., 2015). Among the pro‐inflammatory cytokines analyzed in the hippocampus of aged *Mlkl*‐KO mice only IL‐12 decreased, reaching levels comparable with adult mice (Figure 2). IL‐12 is produced in the brain by microglia and required for IFN‐γ and TNF‐α production, two master inflammatory cytokines. The increased expression of IL‐12 in the brain has been associated with spontaneous neurological disorders in aged mice (Hofer et al., 2004). By contrast, the IL12‐KO mouse exhibits lower levels of microglia activation and reduced neurodegeneration in an excitotoxicity‐mediated injury model (Chen et al., 2004). Additionally, increased levels of the anti‐inflammatory cytokines, IL‐2 and IL‐10, were specifically detected in aged *Mlkl*‐KO mice (Figure 2), suggesting that MLKL could also act as a repressor of anti‐inflammatory cytokine expression under necroptosis activated conditions. Interestingly, IL‐10 inhibits the production of IL‐12 (Lobo‐Silva et al., 2016), which accompanied with the IL‐12 decrease observed in the aged *Mlkl*‐KO mice generates a positive anti‐inflammatory feedback loop, limiting neuroinflammation.

It is well documented that neurodegeneration contributes to cognitive decline during normal aging (Bettio et al., 2017). Our electrophysiological analysis demonstrated that loss of *Mlkl* in aged mice produces changes in the hippocampal circuit that prevent the age‐dependent loss of synaptic strength (Figure 2). The increased facilitation index in aged WT mice is an indicator of a decreased neurotransmitter release probability from the presynaptic compartment. Remarkably, the decreased paired‐pulse facilitation index in aged *Mlkl*‐KO mice indicates that necroptosis is in fact contributing to these axonal defects in aged mice. Electrophysiological results were supported by improved learning and memory performance in aged *Mlkl*‐KO mice, demonstrating that altogether axonal protection, controlled inflammatory status, as well as synaptic transmission restoration in *Mlkl* deficiency favor youthful‐like behavior in aged mice. These observations support the notion that lower levels of necroptosis activation improve key cognitive functions that might positively impact the quality of life, favoring healthy aging.

The brain and peripheral organs share common biological mechanisms of aging (López‐Otín et al., 2013). Thus, the development of anti‐aging drugs that improve cognitive function and hence the quality of life in old age could have a significant potential at improving healthspan. Our pharmacological strategy to inhibit RIPK3 with GSK´872 in aged mice demonstrated to be extraordinarily effective on reverting AxD, neuroinflammation, and hippocampus‐dependent functional impairment (Figure 3). Interestingly, GSK’872 treatment reduced the levels of most of the pro‐inflammatory cytokines analyzed in the hippocampus of aged mice, thereby diminishing neuroinflammation. Moreover, the analysis of serum cytokines showed the same regulatory profile as the brain, demonstrating that systemically, the inflammatory condition of aged mice is maintained in a youthful‐like state under RIPK3 inhibition, reaching levels also comparable to aged *Mlkl*‐KO mice (Figure S8). These results support the emerging idea of considering RIPK3 as a novel master regulator of proinflammatory responses under diverse conditions, including aging (Deepa et al., 2018; Thadathil et al., 2021; Yang et al., 2022) and CNS disorders (Salvadores et al., 2022; Yuan et al., 2018). Therefore, the use of RIPK3 inhibitors stand as a potential therapeutic intervention to inhibit necroptosis and to halt diseases associated with exacerbated or persistent inflammation (Speir & Lawlor, 2021; Xia et al., 2020).

Quantitative proteomic analysis demonstrated that about 7000 proteins changed their expression profile as a consequence of aging (Figure 4 and Figure S12), of which 2516 shown to be upregulated and 2307 were downregulated under necroptosis inhibitory conditions in the hippocampus of aged mice. The pathway analysis unveiled key biological processes with opposed directionality between “normal” and necroptosis‐targeted aging, showing that key hallmarks of aging, including synaptic function, mitochondrial dysfunction, stress response, cellular senescence, deficient nutrient sensing, altered metabolism, and others (Kennedy et al., 2014; López‐Otín et al., 2013), are positively regulated under necroptosis inhibitory conditions. Interestingly, our pharmacological approach demonstrated a similar trend in effect over the proteome as the genetic model, suggesting that necroptosis inhibition could be an attractive therapeutic strategy to slow aging. In fact, results obtained from our proteomic analysis indicated that GSK’872 treatment influences most of the different hallmarks of aging, an inclusion criterion that is currently recognized to accept novel molecule candidates as geroprotectors (Partridge et al., 2020). Furthermore, inhibition of necroptosis modulates several brain functions implicated in brain rejuvenation (Wyss‐Coray, 2016) (Figure 4 and Figure S13), including those elucidated from the proteomic analysis and experimentally evaluated, such as synaptic plasticity and neuronal senescence (Figures 5 and 6). Moreover, other interesting pathways were also demonstrated to be positively modulated by necroptosis inhibition (Figure S15–S18). These pathways include *synaptogenesis signaling*, *calcium signaling*, *CREB signaling in neurons*, and others; most of them highly implicated in the maintenance of neuronal homeostasis and functioning, and consequently in brain‐dependent functions, including cognitive and behavioral processes (Zia et al., 2021).

Overall, our study demonstrates that necroptosis contributes to the age‐associated deterioration of axonal integrity and function, affecting hippocampal synaptic plasticity and neuronal connectivity, and consequently the hippocampal‐dependent cognitive function of aged mice. Our results from the pharmacological intervention indicates that necroptosis inhibition is an interesting and novel therapeutic target to counteract the deleterious effects of aging, thus increasing healthspan and potentially delaying the onset of a range of age‐related disabilities.

## 4 MATERIALS AND METHODS

See Appendix S1 for full experimental procedure.

### 4.1 Animals

Wild‐type (WT) C57BL/6J male mice of different ages were purchase from the Jackson Laboratory and maintained in the Universidad Mayor animal facility. Aging groups were established as follow: adult (3–6 month), old (12–15 month), and aged mice (more than 20 month). The age range for each mice group was selected in equivalence with the human life phases (Flurkey et al., 2007; Hagan, 2017). *Mlkl* knockout mice (*Mlkl*‐KO) (C57BL/6 background) were kindly provided by Dr Douglas Green (St. Jude Children's Research Hospital, Memphis, TN, USA) (Murphy et al., 2013). Genotyping is described as Appendix S1. *Mlkl*‐KO mice were reproduced and bred in the Animal Facility of the Sciences Faculty of the Mayor University. Animals were kept under standard conditions of light and temperature and were feed with food and water ad libitum in the Animal Facility of the Sciences Faculty of the Mayor University. A total of 145 male mice were used to perform the experiments. The research protocol no. 22‐2017 was approved by the Animal Care and Use Scientific Ethic Committee of the Mayor University.

### 4.2 Osmotic pump implantation

Micro‐osmotic pumps (Alzet, model 1004) containing the RIPK3 inhibitor GSK’872 (Tocris) (2 mg/kg) were surgically implanted in the peritoneal cavity of 23‐month‐old mice, previously anesthetized with 2% isofluorane, which was continuously administrated during surgery. The pump allows a constant flux of the drug at 0.11 μL/h for 28 days. One‐month post‐surgery, mice (24‐month‐old) were subjected to behavioral test to evaluate memory and then the tissue was extracted for further analyses.

### 4.3 Immunohistochemistry

Mice were deeply anesthetized with isoflurane and intracardially perfused with isotonic saline followed by 4% paraformaldehyde. Brains were dissected, postfixed overnight in 4% paraformaldehyde at 4°C, and then incubated in 30% sucrose. Tissue was cryoprotected in optimal cutting temperature compound (OCT, Tissue‐Tek) at −20°C, and serial sagittal sections of 20 μm thickness were obtained using a cryostat (Leica, CM1860). Brain sections were pre‐mounted on positively charged slides and washed in TBS. After antigen retrieval (80°C, 30 min, 10 mM citrate buffer, pH 6.0), sections were blocked in TBSB (TBS, 5% BSA and 0.25% Triton X‐100), and then incubated overnight at 4°C with primary antibodies. See Appendix S1 for full experimental procedure.

### 4.4 Image analysis

Confocal images were acquired by confocal laser microscopy with a Leica TCS SP8 microscope with a 40× oil objective NA 1.3, using the following settings: a z‐step size of 0.8 μm optical sections (30 steps), 8‐bit images, 1024 × 1024 pixels, 3× digital zoom, acquisition speed: 200 Hz, unidirectional acquisition without line or frame average. Raw images were exported to the Image J software from the NIH (USA) to generate the maximal projection images. Three‐dimensional projections were created with the Imaris software version x64 9.7.1. Automatic threshold was used to create surfaces for pMLKL, pRIPK3, NF and dapi channels. Phospho‐RIPK3/MLKL colocalization channel was generated using the Imaris Colocalization tool with automatic thresholding. Colocalization analysis was performed with the JACoP plugin (Bolte & Cordelières, 2006). Mander's coefficient M1 was used to estimate the percentage of pRIPK3 signal over pMLKL, while M2 indicates pRIPK3 overlapping with NF‐labeled axons. Axonal degeneration index (DI) was measured in pan‐axonal NF immunostained images as the ratio of the area of fragmented axons against the total axonal area (intact + fragmented particles) in the hippocampus using the particle analyzer algorithm of ImageJ. Fragmented and intact axonal particles were estimated by defining area and circularity of the particles (fragmented: <5 μm^2^ and 0.3 ≤ 1 circularity; intact: ≥5 μm^2^ and 0 < 0.3 circularity) (Arrázola et al., 2019). See Figure S3 for more details.

Axonal pMLKL analysis was performed in hilar fibers by creating a mask from binarized images of the pan‐axonal NF labeling. Threshold was manually defined from the pan‐axonal NF staining in the hippocampus of adult mice and then fixed for all the analyses performed. The mean intensity of pMLKL was analyzed within the axonal mask area. For non‐axonal pMLKL staining, positive cells were counted within a defined area in the hilus and normalized to the total number of cells detected with DAPI.

### 4.5 Learning and memory test

Morris water maze (MWM) navigation task was performed to evaluated spatial memory and learning in adult vs aged WT, *Mlkl*‐KO and GSK’872‐treated mice. Animals were trained in a 1.2‐m‐diameter circular pool (opaque water, 50 cm deep) filled with 19–21°C water. A submerged 11.5‐cm platform (1 cm below the surface of water, invisible to the animal) was used for training, with a maximum trial duration of 60 s; the mice remained on the platform for 10 s at the end of each trial. Each animal was trained to locate the platform for 4 consecutive days following external cues (learning curve), 4 times per day. The test was performed on the fifth day by removing the platform, and swimming was monitored to measure the latency time required to reach the platform and the time spent in each quadrant and in the target quadrant (Q4), and the travelled distance within Q4. Both the learning curve and the test were tracked using an automatic tracking system (ANY‐maze video tracking software, Stoelting Co, Wood Dale, IL, USA) to obtain the parameters measured. Animals with evident swimming difficulties, signs of thigmotaxis or visual defects (such as cataracts) were excluded from the behavioral analysis but also from the biochemical and histochemical analyses.

### 4.6 Senescence‐associated beta‐galactosidase (SA‐βgal) activity

Histochemical detection of SA‐βgal activity was performed as was described before (Debacq‐Chainiaux et al., 2009). Briefly, SA‐βgal activity was determined by incubation with 1 mg/mL of solution of 5‐bromo‐4‐chloro‐3‐indolyl β‐d‐galactopyranoside in 0.04 M citric acid/sodium, 0.005 M K_3_FeCN_6_, 0.005 M K_4_FeCN_6_, 0.15 M NaCl, and 0.002 M MgCl_2_ diluted in phosphate‐buffered saline (pH 6) for 16 h. After the incubation, hippocampal slices were washed with TBS and mounted in superfrost microscope slides (6,776,214; TermoFisher) using Fluoromount‐G (00‐4958‐02; TermoFisher). Images were taken with Nikon Eclipse E200 optic microscope with 4× and 10× objective magnification. ImageJ software was used to process the images. Positive area for SA‐βgal activity was measured and representative images are shown.

### 4.7 Statistical analysis

Animal groups were generated and distributed according an a priori size sample calculation based on LaMorte's Power Calculations, considering a 30% difference in means, 90% power and an alpha level of 0.05. Statistical significance was established at *p* < 0.05 by one‐way ANOVA with Tukey's post‐test or RM‐ANOVA for multiple comparisons. Shapiro–Wilk test was performed for each experimental group to evaluate normal distribution of the values by using the Analyze/Normality Test Tool of GraphPad Prism v8.0.2 software. Data analyses were performed using GraphPad Prism Software v8.0.2 from values corresponding to *n* = 3–6 for image analyses and *n* = 10–19 animals for behavioral tests (precise sample size is detailed in figure legends). Outlier values were identified by applying the “Identify Outliers” in Graphpad Prism software and excluded from statistical analysis (specified in figure captions when corresponds).

### 4.8 Label‐free proteomics

#### 4.8.1 S‐Trap processing of samples

Samples were processed using S‐trap mini protocol (Protifi) (for 310 ug and 110 ug samples) and S‐trap micro protocol (for low conc samples) as recommended by the manufacturer with little modification. Tryptic peptides were pooled, dried, and quantified using Pierce Quantitative fluorometric Peptide Assay (Thermo Scientific). See Appendix S1 for full experimental procedure.

#### 4.8.2 LC–MS methods

Samples were injected onto a nanoscale C18 reverse‐phase chromatography system (UltiMate 3000 RSLC nano, Thermo Scientific) then electrosprayed into an Q Exactive Plus Mass Spectrometer (Thermo Scientific). The data were acquired using a uPAC‐compatible easy spray emitter source operated in positive mode with spray voltage at 2.2 kV, and the ion transfer tube temperature at 275°C. The MS was operated in DIA mode. A scan cycle comprised a full MS scan (*m/z* range from 345 to 1155), with RF lens at 60%, AGC target 3E6, orbitrap resolution 70,000, maximum injection time at 200 ms and source fragmentation disabled. The MS survey scan was followed by MS/MS DIA scan events using the following parameters: collision energy mode set to linear with a normalized HCD collision energy set to 25, orbitrap resolution 17,500, first fixed mass 200 *m/z*, AGC target 3E6, maximum injection time 55 ms, isolation windows were variable from 5 to 66 *m/z*. The inclusion list (DIA windows) and windows widths are shown in Table S2. Data for both MS and MS/MS scans were acquired in profile mode. Mass accuracy was checked before the start of samples analysis.

For data analysis of proteomic data, see Appendix S1.

## AUTHOR CONTRIBUTIONS

MSA, GU, SB, JCC, WC, TMW, and FAC designed experiments, analyzed data, and wrote the manuscript; MSA, ML, GQ, FV, DJL, and HH performed experiments; SI, SLE, and TMW performed bioinformatic and proteomic analysis.

## CONFLICT OF INTEREST STATEMENT

The authors have no conflicts of interest to disclose.

## Supporting information


Appendix S1

## Data Availability

Raw data from Figures 1, 2, 3, 4, 5, 6 are available as an extended excel file named “Figures Raw Data”. Raw data from Figures S1–S10, S14 are available as an extended excel file named “Suppl Figures Raw Data”. The datasets generated from the proteomic analysis will be available via the data repository of Edinburgh University (https://datashare.ed.ac.uk/) with an assigned DOI. All the data that support the findings of this study are also available from the corresponding author upon request.
